# Pharmacokinetics of a single oral administration of two cannabidiol formulations in fed and fasted horses

**DOI:** 10.3389/fvets.2025.1515833

**Published:** 2025-02-19

**Authors:** Alessandra Di Salvo, Marilena Bazzano, Giorgia della Rocca, Roberta Galarini, Andrea Marchegiani, Fabiola Paoletti, Danilo Giusepponi, Matteo Mantovani, Fulvio Laus

**Affiliations:** ^1^Department of Veterinary Medicine, University of Perugia, Perugia, Italy; ^2^CeRiDA - Research Center on Animal Pain, University of Perugia, Perugia, Italy; ^3^School of Biosciences and Veterinary Medicine, University of Camerino, Matelica, Italy; ^4^Istituto Zooprofilattico Sperimentale dell’Umbria e Delle Marche "Togo Rosati", Perugia, Italy; ^5^Farmacia San Carlo, Terre Del Reno (FE), Ferrara, Italy

**Keywords:** cannabidiol, CBD, horse, oral administration, pharmacokinetics

## Abstract

Pain management in horses plays a pivotal role in the therapeutic approach to several diseases. Horses have cannabinoid receptors at the level of dorsal root ganglia, blood vessels, and synoviocytes that can be up or down- regulated by inflammatory conditions, justifying the possible efficacy of exogenous cannabinoids (i.e., phytocannabinoids) in managing several painful pathologies in this animal species. However, the current use of supplements containing cannabidiol (CBD) in equines is based on anecdotal evidence, without the support of sufficient pharmacokinetic studies. In humans, the concentration peak of CBD and the area under the concentration-time curve (AUC) are both strongly influenced by food administration. Also, in equids, the oral bioavailability of some drugs can be influenced by the meal but no information is available about CBD. This study investigated the pharmacokinetics of CBD following single oral administration of two different formulations of pure CBD (oil and paste), dosed at 1 mg/kg, at two different times about food administration. CBD oil and CBD paste were administered orally at 1 mg/kg to eight healthy horses according to a cross over design, and blood samples were taken at pre-fixed time-points for the pharmacokinetic analyses. The obtained pharmacokinetic data did not allow for statistically significant differences between formulations (paste or oil) and feeding time (fed and fasted status). However, following treatment with the paste, the Cmax was achieved in a shorter time range compared to the oily formulation, indicating that it could be a better formulation to consider in future equine studies.

## Introduction

The endocannabinoid system is a sophisticated and intricate signaling network involved in different biological processes, considered to have antioxidant, hypotensive, immunosuppressive, anti-inflammatory, pain-relieving and neuroprotective actions and to play a role in cancer cell proliferation. Moreover, it controls movement, attention, sleep, appetite, learning and memory (1). Recently, the presence of cannabinoid receptors (CB1, CB2, PPAR, TRPV1 and GPR55) was identified in the equine dorsal root ganglia, suggesting a possible role for endocannabinoids in the modulation of pain in this species (2, 3). These receptors are also present in the blood vessels and synoviocytes of the equine metacarpophalangeal joints. Furthermore, it was observed that CB1 and CB2 receptors in synovial cells are up-regulated during inflammation and that CB1 receptors decrease with the increase of osteoarthritis. These features reinforce the hypothesis of a possible role for endocannabinoids in the modulation of articular inflammation and pain (4, 5), and of the possible efficacy of exogenous cannabinoids (i.e., phytocannabinoids) as add-on drugs within multimodal analgesic therapies in managing several painful pathologies in this animal species.

Consequently, supplements containing cannabidiol (CBD) have been made available worldwide for free purchase by horse owners (6). Moreover, CBD can also be used in animals as “a veterinary medicinal product prepared extemporaneously in accordance with the terms of a veterinary prescription” according to the cascade provided by the Regulation (EU) 2019/6 (art. 112, paragraph c) of the European Parliament and of the Council on veterinary medicinal products. Despite the high cost due to the large doses that have to be administered, veterinary practitioners have started to prescrivìbe it for therapeutic purpose in some pathological conditions.

However, CBD use, both as a supplement or as a drug, is based on anecdotal evidence and without the support of pharmacokinetic studies (essential to adopt a rational therapeutic regimen that ensures a drug’s efficacy and tolerability).

In the last few years, several pharmacokinetic studies have been published following oral administration to horses of different CBD-based formulations: CBD/CBD acid derivate (CBDA)-rich hemp oil (7) or pure CBD in soybean (8), sesame (9, 10), sunflower lecithin oil (11) and in micellar formulation (10). In addition, formulations containing the entire phytocomplex, such as hemp pellet (12) and paste (13), were tested. Among all these studies, only two have evaluated the equine oral bioavailability of CBD, which resulted in 8 and 14% following administration of CBD in oil to horses with free access to pasture (8) and in fasted status (10), respectively.

The use of different formulations and types of CBD (pure or containing the entire phytocomplex), as well as the different experimental conditions of the conducted studies (fasted and fed animals, different sampling times, number of samplings), may have influenced the pharmacokinetics of CBD (1). Indeed, these features and the large individual variability in CBD plasma concentrations observed in all cited studies do not allow for identifying a rational dosing regimen in horses.

In a human study, the administration of CBD with a high-fat meal increased both the concentration peak and the area under the concentration-time curve (AUC) by more than four times compared to its administration in fasted status (14). Food is thought to promote the absorption of lipophilic drugs by increasing their time spent in the gastrointestinal tract, their solubilization, and their lymphatic transport by lymphatic lipoproteins (15). It has been observed that the oral bioavailability of drugs can also be influenced by the meal in the equine species (16).

The present project was conducted to evaluate the pharmacokinetics of pure CBD after a single oral administration of two pharmaceutical formulations, paste and oil, administered in fed and fasted horses. The goal was to attest which formulation and feeding condition offered the best pharmacokinetic profile.

## Materials and methods

The study was conducted with the favorable consent of the Local Animal Welfare Committee (OPBA, protocol number E81AC.19) of the University of Camerino and the approval of the Ministry of Health (authorization number: 1021/2023-PR), in accordance with the Directive 2010/63/EU of the European Parliament on the protection of animals used for scientific purpose.

### Animals and treatments

Eight horses (6 females and 2 males), aged between 10 and 15 years, weighing 421.75 ± 95.06 kg (mean ± standard deviation) and considered healthy based on clinical examination, were enrolled in the study. Selected horses were used for pleasure and were therefore accustomed to the presence of humans and to being handled. The anamnestic data of the horses did not report any relevant previous diseases in the last 2 years. The clinical evaluation consisted of a detailed clinical examination of the general conditions such as evaluation of the mucous membranes, lymph nodes, hydration status, cardiovascular, respiratory and digestive systems. Any alteration in the health conditions, monitored during the procedures, would have caused the exclusion of the horse from the study. Written owner consent was obtained for all horses participating in the study.

The horses were housed at the Veterinary Teaching Hospital of the University of Camerino (Italy), stabled in individual boxes but with the possibility of seeing each other to minimize stress factors. They were all fed polyphite hay at a rate of 2.5% of their body weight divided into three daily meals (at 07:00, 13:00 and 19:00), and water was available ad libitum.

A licensed pharmacy prepared both drug galenic formulations containing 20% of pharmaceutical-grade synthetic CBD crystals (purity: 99.4%). CBD oil was obtained by solubilizing CBD in medium-chain triglyceride (MCT) pharmaceutical-grade oil, that was placed in bottles equipped with a graduated dispenser. CBD paste consisted of a base of carboxymethylcellulose and glycerol, with the addition of water, saccharin, apple flavoring, polysorbate as a surfactant (for the solubilization of the CBD), potassium sorbate and sodium metabisulfite as preservatives. It was prepared in individual graduated syringes. The pharmacy indicated the storage conditions and the shelf life of both formulations.

CBD oil and CBD paste were administered orally at 1 mg/kg by inserting the graduated dispenser or syringe into the oral cavity, between the internal surface of the cheek and the premolars, as per everyday veterinary practice for drug administration *per os* in equids. All animals received both formulations 2 h before the morning meal, corresponding to 10 h after the previous meal (fasted status), and 1 h after (fed status). For the treatments, 4 experimental moments were planned, with a washout period of at least 10 days between them: each experimental moment included 2 consecutive days in which 8 horses were treated (four horses per day) according to a cross over design. The treatments scheme and all data on horses are reported as Supplementary materials.

### Blood sampling

Blood samples (10 mL) were taken from the jugular vein following the insertion of a catheter, before drug administration and at pre-fixed time-points during the 24 h following the CBD administration (0.25, 0.5, 0.75, 1, 1.5, 2, 2.5, 3, 4, 6, 8, 10 and 24 h), to determine drug concentration *vs* time curves. The collected blood was placed in tubes containing Na-citrate, and plasma obtained following centrifugation at 1500 rpm for 5 min was frozen and maintained at −80°C until the analysis, carried out within 40 days of collection.

### Analytical determination of CBD

#### Chemical and reagents

Cannabidiol (Cerilliant, Round Rock, TX, United States) and its deuterated analog, cannabidiol-d3 (CBD-d3; Cerilliant) were purchased from Merck (Darmstadt, Germany), as methanolic solutions at concentrations of 1,000 μg/mL and 100 μg/mL, respectively. Working solutions were prepared by diluting stock solutions with MeOH. Acetonitrile and MeOH (LC–MS grade) were obtained from Honeywell (Charlotte, NC, United States), whereas formic acid was purchased from VWR International (Radnor, PA, United States). Dichloromethane was obtained from Carlo Erba Reagents (Cornaredo, MI, Italy) and isopropylic alcohol from Merck. The OASIS HLB cartridges (60 mg/3 mL) were purchased from Waters Corporation (Milford, MA, United States). Water was deionized (>18 MΩ cm^−1^) by a Milli-Q water purification system (Millipore, Bedford, MA, United States).

#### Sample preparation

The sample preparation protocol was optimized starting from methods previously published (12, 17, 18). Two mL of equine plasma were mixed with 5 mL of acetonitrile containing 2 ng of CBD-d3, and after vortexing, samples were placed at −20°C for 10 min. After centrifugation at 4000 rpm for 5 min, the extract volume was reduced to about 1 mL under nitrogen stream (40°C). Then, 500 μL of acetonitrile were added to facilitate CBD solubilization, followed by 10 mL of water. After centrifugation (5 min, 4,000 rpm), the extract was loaded on the OASIS HLB cartridge previously conditioned with 3 mL of MeOH and 3 mL of deionized water. The cartridge was washed with 6 mL of a solution at 25% of MeOH, and then the analyte was eluted with 3 mL of a mixture dichloromethane/isopropyl alcohol 80:20 (v/v). The eluate was evaporated until dryness under nitrogen stream (40°C) and resuspended in 200 μL of MeOH/H_2_O 80:20 (v/v) with 0.1% formic acid. After centrifugation (5 min, 12,000 rpm), the obtained samples were transferred to autosampler vials.

#### LC–MS conditions

Plasma extracts were analyzed using a SCIEX (Framingham, MA, United States) TripleTOF® 6,600+ QTOF coupled with an Exion UHPLC system. Separation was achieved on a Kinetex C8 column (100 × 2.1 mm, 2.6 μm), which was connected to a guard column Kinetex C8 (2.1 × 3 mm), both purchased from Phenomenex (Torrance, CA, United States). The mobile phases were water (A) and MeOH (B), both containing 0.1%. formic acid. The gradient profile was as follows: 0–1 min, 60% B; 1–5 min, to 80% B; 5–8 min, to 100% B; 8–12 min, 100% B; 12–12.1 min, to 60% B, and 12.1–16 min, 60% B. The column temperature was set at 40°C, the flow rate at 0.3 mL/min, and the injection volume was 5 μL. Ionization was achieved in positive mode (ESI+). The source conditions were: ion source gas one 40 psi, ion source gas two 35 psi, curtain gas 45 psi, temperature 500°C, IonSpray Voltage Floating 4,500 V. CBD and CBD-d3 were detected in multiple reaction monitoring (MRM) mode selecting the following transitions: CBD 315.2 *m/z* → 193.1 *m/z*, 315.2 *m/z* → 123.0 *m/z*, 315.2 *m/z* → 259.2 *m/z*; CBDd3 (IS) 318.2 *m/z* → 196.1 *m/z*. Declustering Potential was set at 120 and Collision Energy at 30.

### Pharmacokinetic and statistical analysis

The values of maximum observed CBD plasma concentration (C_max_) and the time this was achieved (T_max_) were directly taken from plasma concentration vs. time curves. The trapezoidal method was used to calculate the areas under the concentration-time curves from 0 to the last time (AUC_0 − t_), while the other main pharmacokinetic parameters were calculated from plasma concentration-time curves obtained for each animal at each treatment by a non-compartmental analysis (NCA) using the PK-Solver program (19).

The normality test to evaluate the distribution of the obtained pharmacokinetic data was executed by the Shapiro Wilk test. The eventual differences in PK parameters between treatments were evaluated by applying the Kruskal-Wallis non-parametric test, considered as more appropriated. All statistical analyses were conducted by Statistics for Data Analysis powered by SPSS version 25 (SPS srl, Bologna, Italy); a *p* value <0.05 was considered to be significant.

## Results

CBD was quantified by applying the isotopic dilution method, using labeled CBD as an internal standard (IS). Four quality control samples (QCs) were inserted in each analytical batch for a total of 24 blank samples, 24 spiked samples at 0.1 ng/mL, 24 at 1.0 ng/mL and twenty-four at 10 ng/mL. Method selectivity was assessed through the 24 blank QC samples. No interfering peaks were detected at the retention times of CBD (6.1 min). Linearity was successfully verified in the concentrations range 1–200 ng/mL (1, 5, 10, 25, 50, 100, 150 and 200 ng/mL of CBD in MeOH/H_2_O mixture 80:20 (v/v) with 0.1% formic acid, each containing 10 ng/mL of IS). Precision and accuracy were assessed by analyzing five replicates of blank plasma samples spiked at four different concentrations (0.1 ng/mL, 0.3 ng/mL, 5 ng/mL, 20 ng/mL) on two different days. The coefficients of variation were estimated both in repeatability (CV_r_) and within-laboratory reproducibility (CV_wR_) conditions using ANOVA. Table 1 reports the obtained values. The lower limit of quantification (LLOQ) was fixed at the first validation level (0.1 ng/mL). The matrix effect was not significant, ranging from 90 to 110%. Samples with concentrations higher than 20 ng/mL were afresh extracted, introducing an appropriate dilution factor and reanalyzed.

**Table 1:** Precision and accuracy data of analytical method at the four validation cannabidiol concentrations.

CBD Concentration (ng/mL)	CV_r_ (%)	CV_wR_ (%)	Accuracy (%)
0.1	8.4	8.4	98 ± 8
0.3	3.7	7.0	103 ± 6
5	2.3	2.3	116 ± 2
20	1.7	2.8	111 ± 2

CV_r_ and CV_wR_ are the coefficient of variations estimated in repeatability and within-laboratory reproducibility conditions, respectively.

Thanks to the graduated dispensers, both formulations allowed for an easy (small volume of drug, from 1.5 to 2.9 mL) and precise (between 0.99 and 1.01 mg/kg b.w.) dosing of CBD. The treatments resulted well tolerated and no adverse events were observed.

**Figure 1:**
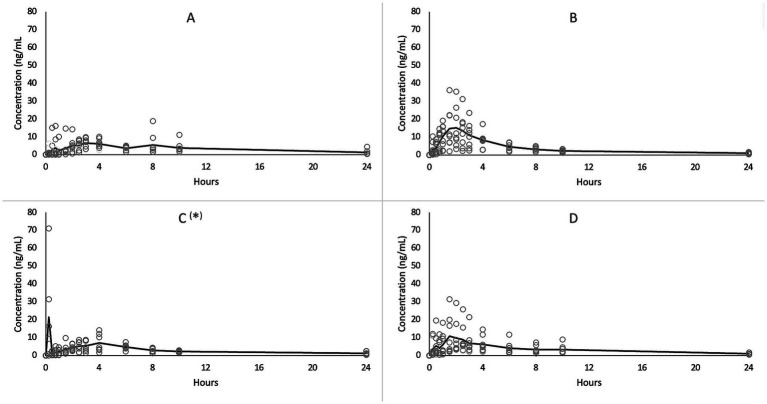
Average (solid line) and single (circles) CBD plasma concentrations versus time following single oral administration of oil **(A,C)** and paste **(B,D)** in 8 horses in fasted **(A,B)** and in fed status **(C,D)**.

The plasma CBD concentrations vs. time revealed a large individual variability following all treatments, as depicted in Figure 1.

**Figure 2:**
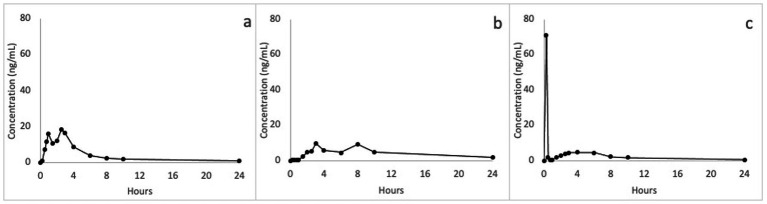
Example of profiles plasma concentrations versus time, following single oral administration of 1 mg/kg of CBD in horses, where evident fluctuations in concentration were observed. **(a)** CBD paste in fed status; **(b)** CBD oil in fasted status; **(c)** CBD oil in fed status.

Multiple peaks during the first 4 h following treatments were detected in some horses. In addition, little fluctuations in CBD plasma concentrations were sometimes observed in the terminal phase (between 8 and 10 h) of the concentration-time curves; these fluctuations were generally lower than 0.5 ng/mL, with only one fasted horse showing a secondary peak similar to the C_max_ at 8 h following CBD oil administration. In Figures 2A,B, two examples of concentration-time profile with a secondary peak are reported.

Following paste administration, CBD was quantifiable from the first to the last sample point in all horses, both in fed and fasted conditions. In fasted horses, C_max_ was achieved between 1.50 and 3 h following CBD administration, while in fed horses C_max_ was observed at the first sampling time (0.25 h) in two subjects and between 1.50 and 4 h in the remaining animals. The C_max_ ranged from 3.32 to 36.23 and from 3.45 to 31.4 ng/mL in fasted and fed status, respectively.

After treatment with oil in fasted condition, CBD was quantifiable from 15 min following administration in seven horses, while in the remaining horse it was detectable from 45 min and quantifiable from 1.5 h. In all animals, CBD continued to be quantifiable for up to 24 h. The C_max_ was achieved at 3 h in 3 horses, with the other horses showing the C_max_ at a different times between 0.25 and 8 h. The obtained range of C_max_ was between 6.19 and 18.90 ng/mL.

Following administration of CBD oil in the fed condition, one horse showed very low plasma CBD concentrations, close to the LOQ value (0.1 ng/mL), up to 8 h, and less than 0.8 ng/mL in the remaining two sample times; a possible explanation may be the oil leakage through salivation and head movements. This concentration-time curve was thus excluded from the results analysis. Three horses showed a C_max_ values from 16.2 to 71.1 ng/mL at 15 min following CBD administration, while the remaining animals showed a C_max_ ranging from 5.53 to 14.2 between 1.5 and 6 h. Figure 2C reports the concentrations – time profile of the horse with the C_max_ of 71.1 ng/mL. It is possible to observe that at 45 min after treatment, CBD plasma concentrations dropped to a value close to the LOQ value, rising again at 2.5 h at concentrations above 4 ng/mL, which were maintained up to 6 h.

The NCA was not performed for two horses treated with CBD oil in fasted condition (the one with the C_max_ at 8 h and the horse displaying a secondary peak at 8 h with a concentration value similar to the C_max_), because there were not enough points in the terminal phase (at least 3 points after C_max_ needed). In two fed horses treated with CBD oil and one fed horse treated with the paste, the terminal phase obtained from the NCA was considered unreliable by virtue of an extrapolated AUC percentage greater than 46%. For these subjects, only the C_max_, T_max_ and AUC_0-t_ were considered.

Table 2 reports the main pharmacokinetic parameters obtained from the CBD concentration vs. time curves following CBD administration both as oil and paste at the two feeding conditions. The data were generally normally distributed; thus, they are reported as mean ± standard deviation (S.D.).

**Table 2:** Main pharmacokinetic parameters obtained following oral administration of CBD oil or paste at 1 mg/kg in 8 horses both in fed and in fasted status (Data expressed as mean ± S.D. and range in brackets).

PK Parameter		CBD treatment			
	Unit	Oil in fasted status	Paste in fasted status	Oil in fed status	Paste in fed status
t_1/2λz_	h	6.70^§^ ± 0.79* (5.58–7.77)^(b)^	8.36^§^ ± 1.59* (6.70–12.91)	7.76^§^ ± 3.63* (4.56–11.39)^(a)^	7.20^§^ ± 2.49* (4.39–10.11)^(c)^
T_max_	h	2.50 ± 2.51 (0.25–8)	1.94 ± 0.56 (1.50–3)	2.32 ± 2.34 (0.25–6)	1.75 ± 1.25 (0.25–4)
C_max_	ng/mL	12.12 ± 4.63 (6.19–18.90)	17.35 ± 10.77 (3.32–36.23)	22.91 ± 22.76 (5.53–71.10)	14.18 ± 8.53 (5.28–31.35)
AUC _0-t_	ng/mL*h	80.55 ± 44.58 (35.61–177.96)	88.72 ± 40.65 (37.39–161.49)	68.81 ± 14.23 (51.77–95.31)	81.15 ± 51.38 (32.53–177.23)
AUC _0-∞_	ng/mL*h	65.42 ± 17.77 (40.21–89.19)^(b)^	100.46 ± 45.93 (41.59–180.77)	80.53 ± 23.54 (54.14–117.32)^(a)^	95.14 ± 56.58 (37.10–196.65)^(c)^
AUC_extrap_	%	7.91 ± 2.70 (4.68–11.43)^(b)^	11.50 ± 4.30 (5.29–17.76)	11.52 ± 5.62 (4.38–18.76)^(a)^	9.91 ± 5.54 (3.16–17.42)^(c)^
AUMC	ng/mL*h^2^	599.40 ± 165.37 (437.58–857.62)^(b)^	996.37 ± 472.58 (374.84–1637.70)	896.48 ± 454.95 (504.42–1630.69)^(a)^	912.54 ± 472.52 (409.68–1796.04)^(c)^
MRT	h	9.30 ± 1.51 (6.70–10.88)^(b)^	10.02 ± 2.06 (6.80–12.74)	10.66 ± 2.27 (7.88–13.90)^(a)^	10.06 ± 1.61 (8.67–13.30)^(c)^

AUC0-t: area under serum concentration-time curve from zero up to the last concentration ≥ LOQ; AUC0-∞: area under serum concentration-time curve from time zero to infinity; AUCextrap%: Area under the concentration-time curve extrapolated from t to ∞ in % of the total AUC; AUMC: Area under the first moment of the concentration-time curve; Cmax: Maximum concentration observed; MRT: mean residence time; Tmax: Time of maximum concentration observed; t_1/2λz_: terminal half-life. S.D., standard deviation. ^§^Harmonic mean. *pseudo standard deviation.

(a) Calculated on 5 horses.

(b) calculated on 6 horses.

(c) calculated on 7 horses.

No significant difference was observed in pharmacokinetic parameters among different groups of treatment.

## Discussion and conclusion

This study investigated the pharmacokinetics of CBD of two different formulations (oil and paste) of pure CBD, dosed at 1 mg/kg, at two different times about food administration (fasted and fed horses), following oral administration by a graduated dispenser (for oil) or syringe (for paste).

For this purpose an analytical method was developed in order to achieve LLOQs in the order of sub-ppb. Starting by the our previous study on dogs (20), the sample preparation protocol was modified reconcentrating 10 fold horse plasma thanks to the insertion of a clean-up step by means of solid-phase extraction (SPE). The validation data in Table 1 were satisfactory with good precision and accuracy also at the first tested concentration, 0.1 ng/mL, which can be, therefore, chosen as LLOQ.

The drug was administered for either formulation at 1 mg/kg based on the dosage found to be effective following oral administration in the only two clinical cases reported in the literature (21, 22). In the first case report, a horse suffering from mechanical allodynia was orally treated with CBD at 0.5 mg/kg twice daily (21); in the second, the same dosage was used to resolve chronic crib-biting and wind-sucking (22). As the present study provided a single CBD administration, the whole daily dose (1 mg/kg) was administered.

By virtue of its lipophilicity, CBD is considered better absorbable when formulated in a lipidic medium as oil (10). The choice to use MCT oil was due to its ability to prevent the oxidative degradation of CBD and its flavourlessness (23). On the other hand, the concomitant evaluation of a paste formulation was dictated by a wide use of this type of formulation in equine clinical practice due to its ease of administration (16) and the fact that the paste could potentially reduce any losses resulting from the oral administration of liquid formulations due to high salivation [above 20 L/day (24)] and head movements.

In this study, horses were compliant with the administration of both formulations, and no adverse events were observed. This is in line with previous studies, where CBD was well tolerated in horses following a single oral administration at the dose ranging from 0.2 to 8 mg/kg (7, 9, 13).

As observable in Figure 1, the present study showed a large variability in CBD plasma concentrations among horses. The same phenomenon was previously reported following oral CBD administration not only in horses (7, 9–11, 13) but also in other animal species such as dogs, cats, rabbits, parrots, and calves (25).

The mean plasma C_max_ values of CBD observed following administration of both formulations and at both feeding times were at least twice higher than those reported in previous studies after oral administration of CBD at the same dose in horses (9, 11, 13). This difference could be related to different CBD types (purified, full spectrum or synthetic) and formulation (paste, oil, pellet), as well as to the different sampling times and LOQ of analytical methods used in the cited studies; all these differences make not easy to compare the results among them.

Surprisingly, a maximum concentration of 71.10 ng/mL was observed at 15 min from the administration of the oily formulation in one fed horse. This value is much higher than all C_max_ values observed in this study. To explain this high concentration peak, it is conceivable that a large part of the administered dose was absorbed by the oral transmucosal route, reaching the buccal veins that converge in the jugular vein: the blood collection from this site could have led to overestimated CBD plasma concentrations. Indeed, in a previous study, after sublingual administration of 40 μg/kg of detomidine as oromucosal gel in horses, a higher C_max_ was observed after blood sampling from the jugular compared to the lateral thoracic vein (161vs 4.16 ng/mL) (26, 27). Hedges and co-workers further demonstrated the impact of the choice of blood collection vein on drug concentrations following a transmucosal absorption, confirming the jugular vein as a not acceptable sampling site because of observed overestimated drug concentrations (28). The eventual transmucosal absorption of CBD could be avoided by administering the oil by nasogastric tube; however, this procedure does not reproduce the administration in normal field conditions (29). Moreover, the administration by nasogastric tube, often used in equine pharmacokinetic studies, would not have been suitable in the present study due to the tiny amount of administered oil (< of 3 mL) that could have remained attached to the tube (generally long approximately 2 meters) also flashing the tube with a large volume of water by virtue of its lipophilicity.

The transmucosal absorption of a portion of CBD could also have been one of the causes of the fluctuations of CBD concentrations observed in this study. The existence of more than one site of drug absorption is one of the reasons for multiple peaks in the concentration-time profile of a drug, together with the enterohepatic cycle, delayed gastric emptying, interaction with feed and factors linked to formulation (30). Secondary peaks were also observed in dogs after administration of different CBD oral formulations (31, 32) and in the plasma profile of CBDA and THCA following oral administration of a CBD/CBDA-rich hemp oil to horses (7). Double peaks are considered to incur commonly in horses after oral drug administration (7).

It has been hypothesized that fibers such as hay could adsorb some drugs, thus delaying their oral absorption because they need to reach the cecum and be released by the digestive processes (29). In this study, no significant differences were observed in T_max_ between fed and fasted horses. However, it is not possible to exclude that the observed great variability intra- and inter-groups could have masked a difference.

While in a human study, a high-fat meal caused a four-fold increase of C_max_ and AUC values compared to those obtained in fasted conditions (14), in the present study, the feeding status did not influence the C_max_ and AUC values of both CBD formulations. It should be underlined that horses were fed with a polyphite hay, with a low fat content; consequently, no CBD solubilization and no lymphatic absorption related to the lipid content of the meal could have been promoted (15).

Similarly to the present study, a “food effect” on CBD AUCs was not evidenced in dogs (33), while it was instead observed in cats and rabbits (higher and lower AUC in fed conditions, respectively) (34, 35).

Another possible reason for the lack of significant differences between the two feeding conditions and the two formulations could be related to a type II error (i.e., the difference exists, but it is not detected due to the small number of subjects enrolled in the study); however, a minimum of 5 or 6 horses is usually considered acceptable for a crossover pharmacokinetic study (36). A study on a larger number of animals would certainly be more valuable, given the intraspecific variability.

In the present study, the obtained pharmacokinetic data did not allow for a statistically significant difference between formulations (paste or oil) and feeding time (fed and fasted status). However, following treatment with the paste formulation, the Cmax was achieved in a shorter range of time compared to the oily formulation, indicating that it could be a better formulation to consider in future equine studies. Unfortunately, since to the authors’ knowledge the plasma minimum effective concentration of CBD has not yet been defined in horses or in any other animal species, it is not possible to speculate on the achievement of the effective blood concentrations and on the possible duration of action of the drug.

Due to CBD’s potential therapeutic use in horses for orthopedic pain management (37, 38), behavioral disorders (21, 39), mechanical allodynia (20) and dermatologic diseases (40), further studies are warranted to explore the best formulation that assures a more uniform pharmacokinetic profile of CBD.

## Data Availability

The raw data supporting the conclusions of this article will be made available by the authors, without undue reservation.
