# Supplementary material for: Pharmacokinetics of a single oral administration of two cannabidiol formulations in fed and fasted horses
Source: Front Vet Sci. 2025 Feb 19;12:1515833. doi: 10.3389/fvets.2025.1515833 (PMC11880937; doi:10.3389/fvets.2025.1515833)
Supplement: Supplementary file 1 [file Table_1.DOCX]

Supplementary Material

Age, sex, weight and mL of CBD formulation administered

| Horse | Age  (Years) | Sex | Weight  (kg) | Volume administered  (mL) |
| --- | --- | --- | --- | --- |
| A | 10 | F | 400 | 2 |
| B | 15 | F | 317 | 1,6 |
| C | 15 | M | 455 | 2,3 |
| D | 10 | F | 580 | 2,9 |
| E | 15 | F | 483 | 2,4 |
| F | 14 | F | 360 | 1,8 |
| G | 13 | M | 479 | 2,4 |
| H | 12 | F | 300 | 1,5 |

Scheme of treatments

| Horse | Administration | | | |
| --- | --- | --- | --- | --- |
|  | 1^st^ | 2^nd^ | 3^th^ | 4^th^ |
| A | X | Y | Z | W |
| B | X | W | Z | Y |
| C | Z | Y | X | W |
| D | Z | W | X | Y |
| E | Y | X | W | Z |
| F | Y | Z | W | X |
| G | W | X | Y | Z |
| H | W | Z | Y | X |

X: CBD oil in fasted horse

Y: CBD oil in fed horse

Z: CBD paste in fasted horse

W: CBD paste in fed horse
